# Productive Hepatitis C Virus Infection of Stem Cell-Derived Hepatocytes Reveals a Critical Transition to Viral Permissiveness during Differentiation

**DOI:** 10.1371/journal.ppat.1002617

**Published:** 2012-04-05

**Authors:** Xianfang Wu, Jason M. Robotham, Emily Lee, Stephen Dalton, Norman M. Kneteman, David M. Gilbert, Hengli Tang

**Affiliations:** 1 Department of Biological Science, Florida State University, Tallahassee, Florida, United States of America; 2 Department of Biochemistry and Molecular Biology, University of Georgia, Athens, Georgia, United States of America; 3 Division of Transplantation, Department of Surgery, University of Alberta, Edmonton, Alberta, Canada; University of Kentucky College of Medicine, United States of America

## Abstract

Primary human hepatocytes isolated from patient biopsies represent the most physiologically relevant cell culture model for hepatitis C virus (HCV) infection, but these primary cells are not readily accessible, display individual variability, and are largely refractory to genetic manipulation. Hepatocyte-like cells differentiated from pluripotent stem cells provide an attractive alternative as they not only overcome these shortcomings but can also provide an unlimited source of noncancer cells for both research and cell therapy. Despite its promise, the permissiveness to HCV infection of differentiated human hepatocyte-like cells (DHHs) has not been explored. Here we report a novel infection model based on DHHs derived from human embryonic (hESCs) and induced pluripotent stem cells (iPSCs). DHHs generated in chemically defined media under feeder-free conditions were subjected to infection by both HCV derived in cell culture (HCVcc) and patient-derived virus (HCVser). Pluripotent stem cells and definitive endoderm were not permissive for HCV infection whereas hepatic progenitor cells were persistently infected and secreted infectious particles into culture medium. Permissiveness to infection was correlated with induction of the liver-specific microRNA-122 and modulation of cellular factors that affect HCV replication. RNA interference directed toward essential cellular cofactors in stem cells resulted in HCV-resistant hepatocyte-like cells after differentiation. The ability to infect cultured cells directly with HCV patient serum, to study defined stages of viral permissiveness, and to produce genetically modified cells with desired phenotypes all have broad significance for host-pathogen interactions and cell therapy.

## Author Summary

Physiologically relevant cell-culture models for infection with hepatitis C virus (HCV) are scarce, and infection by viruses derived from patient serum has been inefficient. Differentiated human hepatocyte-like cells derived from pluripotent stem cells demonstrate hepatic functions but have not been explored for HCV infection studies. Here we report a novel infection model based on these hepatocyte-like cells. Stem cells and definitive endoderm successfully resisted HCV infection, whereas hepatic progenitor cells derived from the stem cells were productively infected by both human- and cell-culture-derived HCV. We determined the point of transition from resistance to susceptibility and, by comparative gene profiling, identified the host factors that were correlated with susceptibility. Genetic modification of human embryonic stem cells, coupled with hepatic differentiation, generated hepatocyte-like cells that were resistant to HCV infection. Our study establishes a new noncancerous and renewable cell-culture system for HCV infection, permits direct infection of cells by patient sera in vitro, identifies a defined transition to HCV susceptibility during hepatocyte differentiation, and demonstrates the feasibility of generating virus-resistant human hepatocyte-like cells in vitro.

## Introduction

Chronic infections by hepatitis viruses such as hepatitis B virus (HBV) and hepatitis C virus (HCV) afflict more than 550 million people worldwide and cause serious liver diseases such as cirrhosis and hepatocellular carcinoma (HCC) [1], [2]. These end-stage diseases destroy the self-regenerating ability of the organ and commonly require liver transplantation for patient survival. Unfortunately, in addition to the issue of donor shortage, HCV-related liver-transplant patients, who account for almost half of those on the waiting list, are confronted by the serious problem of reinfection of the new graft. The current reinfection rate is 100%, and disease progression appears to be accelerated with posttransplant reinfection [3]. An alternative to solid organ liver transplant is hepatocyte transplantation, which could help alleviate the shortage of donor organs [4] and might allow blockade of reinfection if the hepatocytes could be made resistant before engraftment. Studies with immunodeficient mouse models indeed demonstrated that purified primary human hepatocytes (PHHs) could repopulate damaged mouse liver after transplantation [5]–[7]. Obtaining sufficient numbers of genetically modified PHHs has not been possible, however, as these cells do not readily proliferate ex vivo, so their expansion and genetic modification are restricted. In addition, uninfected PHHs will necessarily be from a different individual than the recipient, presenting the risk of transplant rejection as in the case of solid liver transplantation.

PHH cultures, established from adult or fetal livers, also represent the most physiologically relevant target cells for HCV infection in vitro. Despite the popularity and success of the cell-culture system based on the hepatoma cell line Huh-7 and its derivatives [8], [9], several important aspects of viral infection and host responses cannot be studied in these cell lines. For example, the highly permissive Huh-7.5 cells are defective in RIG-I-mediated interferon production [10] and therefore not suitable for studies of innate immunity to HCV infection. Cell lines outside the Huh-7 series that can support HCV infection have been also reported [11]–[15], but in addition to having much lower infection efficiencies, these cells are either derived from tumor tissues or immortalized, making them incompatible with any research intended to determine potential oncogenic effects of viral infection. Notwithstanding the importance of PHHs, the usefulness of these cells as a robust culture model for HCV research has been significantly limited by poor accessibility and lot-to-lot variability. Procurement of liver biopsy and freshly isolated hepatocytes is difficult for the majority of the researchers, and the commercial supplies of PHHs can be unpredictable because of the low plating efficiency of the cells. The variability of PHHs isolated from different patients is another challenge. Differences in patient medical history, host genetics, and methods of isolation all contribute to the difficulty of obtaining reproducible results and comparing data from different labs. For example, Podevin et al. [16] noted that PHH cultures established from patients who had a history of heavy alcohol use were not suitable for infection by HCV produced in cell culture (HCVcc). Finally, in studies of interferon (IFN) production in response to HCV infection where experiments cannot be performed with Huh-7.5 cells, special care has to be taken to eliminate the potential co-purification of nonparenchymal cells from liver tissue as those can complicate results regarding the cellular source for IFN production [17].

The source of infectious HCV particles that can be used in infection studies in cell culture is also limited. The discovery of a genotype 2a genome (JFH-1) that could replicate in cell culture without adaptive mutations [18] led to the production of infectious HCVcc particles [19]–[22], now ubiquitously used in cell-culture experiments. These JFH-1–based viruses, along with additional chimeras [23], [24] and a genotype 1a virus that could also produce particles when adaptive mutations were introduced into its genome [25], greatly advanced the cell culture model beyond the subgenomic replicon stage and allowed studies of the full life cycle of HCV. Nevertheless, HCV particles derived from patient serum (HCVser) may differ from HCVcc in important aspects such as buoyant density and virion-associated serum products that are only present in vivo. HCVser infection in vitro has been inefficient, and a recent study with the human liver progenitor cell line HepaRG suggests that both immature and mature hepatocyte features are required for efficient infection and replication of HCVser [12].

Emerging stem cell technologies may offer an elegant solution to these problems. Pluripotent stem cells, either embryonic or induced by reprogramming factors (hESCs and iPSCs, respectively), have the remarkable ability of indefinite self-renewal while maintaining their potential to differentiate into virtually any cell type [26], [27], including hepatocyte-like cells [28]–[35]. In vitro differentiated human hepatocyte-like cells (DHHs) express hepatic markers and display hepatic function. More importantly, DHHs were able to repopulate mouse liver and exhibit hepatic function after transplantation in a liver-damaged mouse model [36]. Combining genetic manipulation of pluripotent cells with directed hepatic differentiation holds great promise for generating virus-resistant hepatocytes to be used in a potential life-saving therapy, but whether DHHs can be productively infected by HCV has not been studied, so their utility in the setting of HCV-related hepatocyte transplantation has not been explored. Here we report a proof-of-concept study designed to investigate the permissiveness of DHHs to HCV infection as well as the feasibility of genetically modifying pluripotent stem cells and the resulting DHHs to render them resistant to HCV infection. We demonstrated that DHHs derived from both hESCs and iPSCs could be persistently infected with both HCVcc and HCVser, and knocking down critical cellular cofactors for HCV replication [37] in the stem cells before hepatic differentiation generated hepatocytes that were refractory to HCV infection. We also discovered a critical transition stage at which the differentiated cells became susceptible to HCV infection, revealing a mechanism of HCV's tropism for hepatocytes; and potentially exposing additional vulnerabilities of the virus.

## Results

### In vitro differentiated hepatocytes derived from either hESCs or iPSCs are permissive to HCV infection

**Figure 1:**
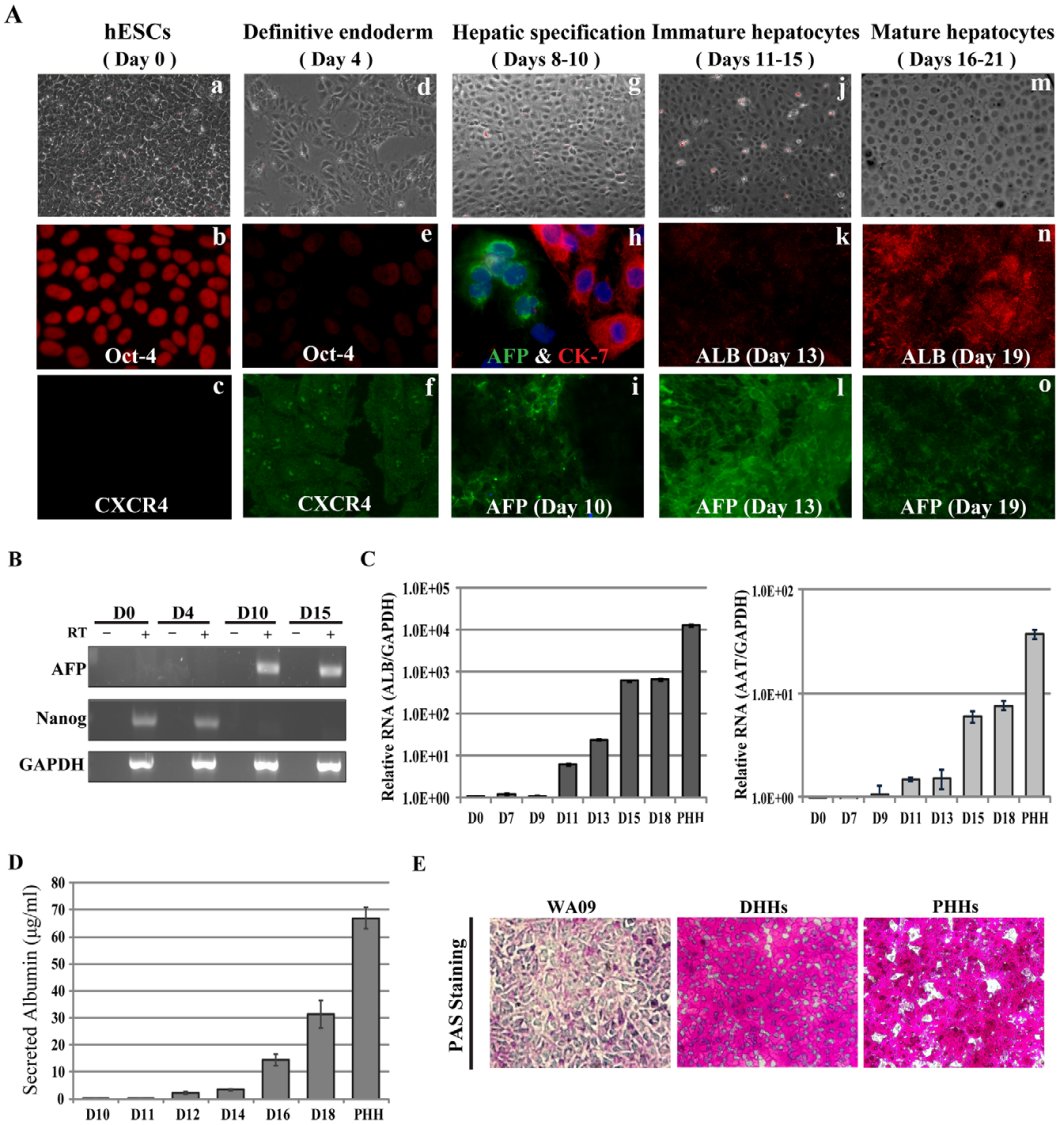
Hepatic differentiation from human embryonic stems cells (hESCs). (*A*) Representative images of cell morphology and protein marker expression of hESCs (day 0), definitive endoderm (day 4), hepatic progenitor cells (days 8–10), and hepatocyte-like cells (both immature and mature, days 11–21). For day-10 cells, double-staining of AFP and CK-7 (middle panel, 40×) showed mutually exclusive expression in the cell population. (*B*) Reciprocal expression of pluripotent marker Nanog and liver-specific marker AFP during differentiation. RT: reverse transcriptase. (*C*) Expression of mRNAs of ALB and AAT during differentiation. PHH: primary human hepatocytes; (*D*) Albumin secretion by differentiated human hepatocyte-like cells (DHHs). Culture media were collected at the indicated time points during differentiation and subjected to albumin detection with an ELISA kit. Error bars represent standard deviation from replicate experiments. (*E*) Periodic acid-Schiff staining of stem cells (WA09), DHHs, and PHHs.

We first determined whether DHHs derived from directed differentiation of hESCs or iPSCs were susceptible to infection by HCVcc. A serum-free protocol based on chemically defined culture media [32], [38] was used to differentiate the hESC line WA09 (H9) [26] or the iPSC line (iPS.K3) [31] into hepatic lineage cells that expressed various hepatic markers at different stages of differentiation (Figure 1A; Figure S1 in Text S1). The expression of a pluripotency marker, Oct 4, was high in stem cells but decreased in the definitive endoderm (day 4), whereas the endoderm marker CXCR4 exhibited the reciprocal expression pattern (Figure 1A, panels a–f). The mRNA level of another pluripotency marker, Nanog, also decreased at day 4 and became undetectable at later days (Figure 1B). At day 10 after differentiation, the cells were positive for either alpha-fetoprotein (AFP) or cytokeratin-7 (CK-7) but not both, a pattern suggesting that they are of a composition similar to that of the bipotent hepatoblasts (Figure 1A, panel h); AFP expression steadily increased in the next five days from 5% at day 10 to over 90% at days 13–16. The intensity of AFP staining then decreased when albumin (ALB) started to be expressed in approximately half of the cells towards the end of the differentiation protocol (Figure 1A, panels j–o). Quantitative reverse-transcriptase coupled PCR (qRT-PCR) confirmed that the ALB mRNA continuously increased during differentiation, as did the alpha-1 antitrypsin (AAT) mRNA (Figure 1C). Secretion of ALB into culture medium was evident from day 12 after differentiation and highest after 18 days (Figure 1D). Finally, Periodic acid-Schiff staining revealed that over 80% of the cells at day 18 were capable of glycogen storage (Figure 1E).

**Figure 2:**
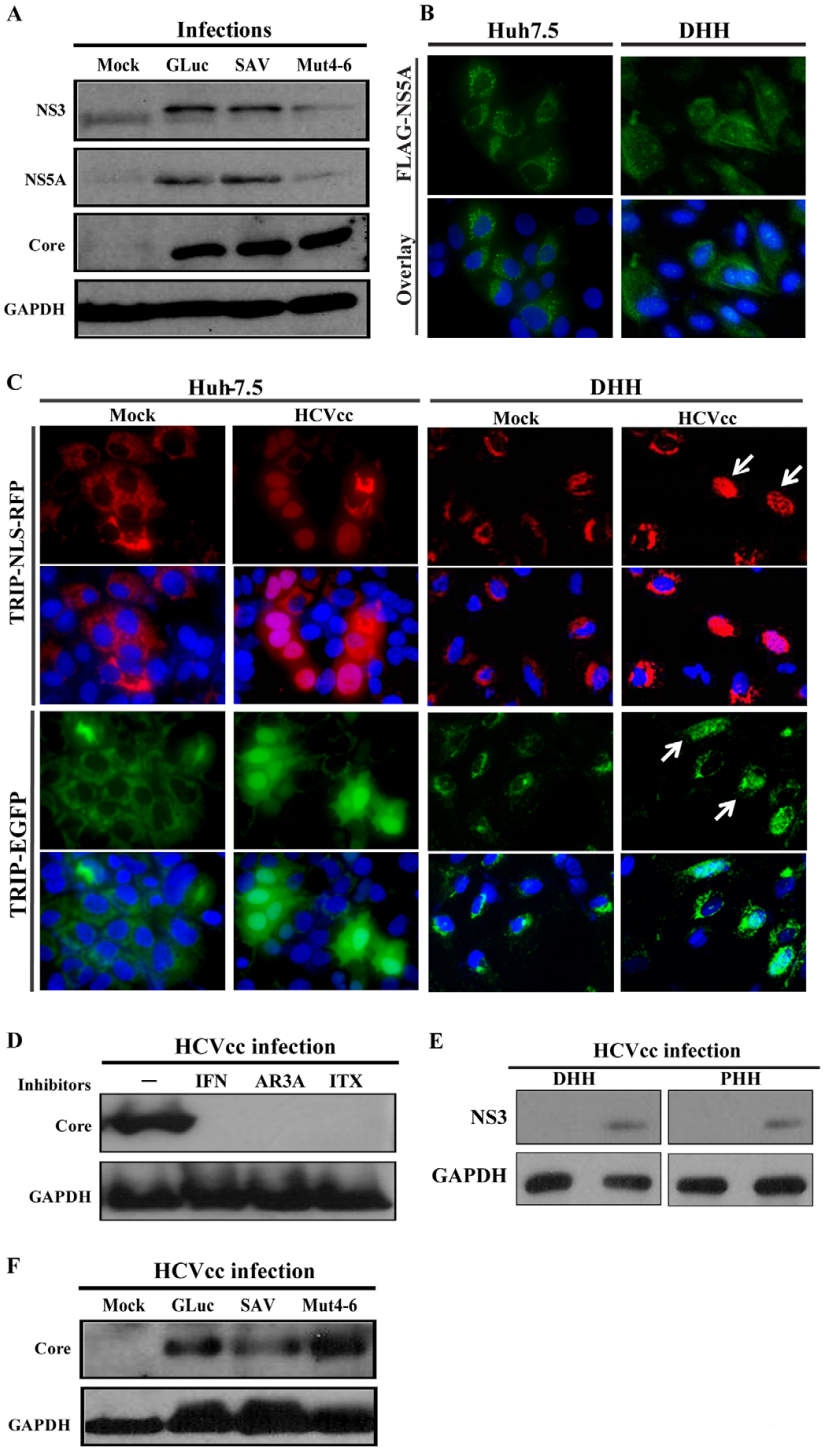
Infection of DHHs derived from hESCs and induced pluripotent stem cells (iPSCs). (*A*) Detection of hepatitis C virus (HCV) proteins in DHHs infected with JFH-1 based HCV derived in cell culture (HCVcc). DHHs were inoculated with three different preparations of HCVcc at day 13 after differentiation, and cell lysates collected at day 21 for western-blot analysis. The anti-NS3 antibody also recognized a nonspecific band in the mock-infected sample. (*B*) Immunostaining of infected DHHs. A JFH variant containing a FLAG tag in the NS5A protein was used to infect either Huh-7.5 or DHHs, and staining was done with an anti-FLAG antibody. (*C*) Infection of Huh-7.5 and DHHs as measured by the HCV-dependent fluorescence relocalization assay. Reporter-transduced cells were infected with HCVcc, and the cells were fixed for immunofluorescence analysis 72 h after infection. For the RFP-NLS-IPS expressing cells, HCV infection led to complete nuclear translocation of the RFP; for the EGFP-IPS cells, HCV infection led to redistribution of green fluorescence from a reticulate cytoplasmic pattern to a diffused pattern with nuclear enrichment. (*D*) HCV inhibitors abolished infection in DHHs. The following inhibitors were included in the infection experiments. IFN: interferon-α, 80 units/ml; AR3A: anti-E2 neutralizing antibody, 1 µg/ml; ITX: ITX5061, an SR-BI inhibitor, 1 µM. (*E*) Comparison of HCVcc infection levels in DHHs and primary human hepatocytes. Primary human hepatocytes were infected for 8 days, for comparability with the DHHs, which were infected at day 13 and the lysed at day 21. (*F*) Infection of DHHs derived from an iPSC line. Differentiation and infection of iPS.K3 were performed as described for H9-derived DHHs (Figures 1 and 2A).

We used three distinct variants of JFH-1 for the initial infection at day 13 and then collected cell lysates at the end of the differentiation period (day 21) for western blotting to detect HCV protein expression. The multiplicity of infection (m.o.i.) used was 0.5. Two of the JFH-1 genomes contained adaptive mutations that increased their infectious titers by at least 100-fold over the JFH-1 wildtype (wt) background. Mut4-6 has been reported previously [39] and the serially adapted virus (SAV) was obtained by repeated passage of JFH-1 HCVcc in Huh-7.5 cells. The third HCVcc variant is Jc1/GLuc2A, a J6/JFH chimera with a *Gaussia* luciferase (GLuc) reporter gene incorporated [40]. Expression of HCV proteins, core, NS3, and NS5A were readily detected by western blotting for all three HCVcc preparations (Figure 2A). Intracellular expression of HCV antigen was also detectable by immunofluorescent staining after infection by a fourth JFH-1 variant that encoded a FLAG-tagged NS5A (Figure 2B). In addition, we confirmed infection events in single cells by introducing an HCV-dependent fluorescence relocalization (HDFR) reporter construct [41] into the day-10 cells and monitoring the nuclear translocation of a fluorescent protein upon cleavage of its mitochondria anchor by the HCV NS3 protease (Figure 2C). To determine whether HCVcc infection of DHHs depended on viral glycoproteins and cell-surface receptors, we performed the infection in the presence of a neutralizing E2 antibody [42] and a small-molecule compound that inhibits the scavenger receptor class B type I (SR-BI) binding [43]. Both agents efficiently blocked infection, as did the replication inhibitor IFN-α (Figure 2D). A comparison of HCV expression levels in similarly infected DHHs (WA09-derived) and PHHs (isolated from a patient) revealed that efficiency of infection in DHHs is comparable to that in PHHs (Figure 2E). Finally, DHHs derived from an iPSC cell line (iPS.K3) also supported robust infection by all three derivatives of the JFH-1/HCVcc (Figure 2F).

### DHHs support persistent infection and produce infectious particles

**Figure 3:**
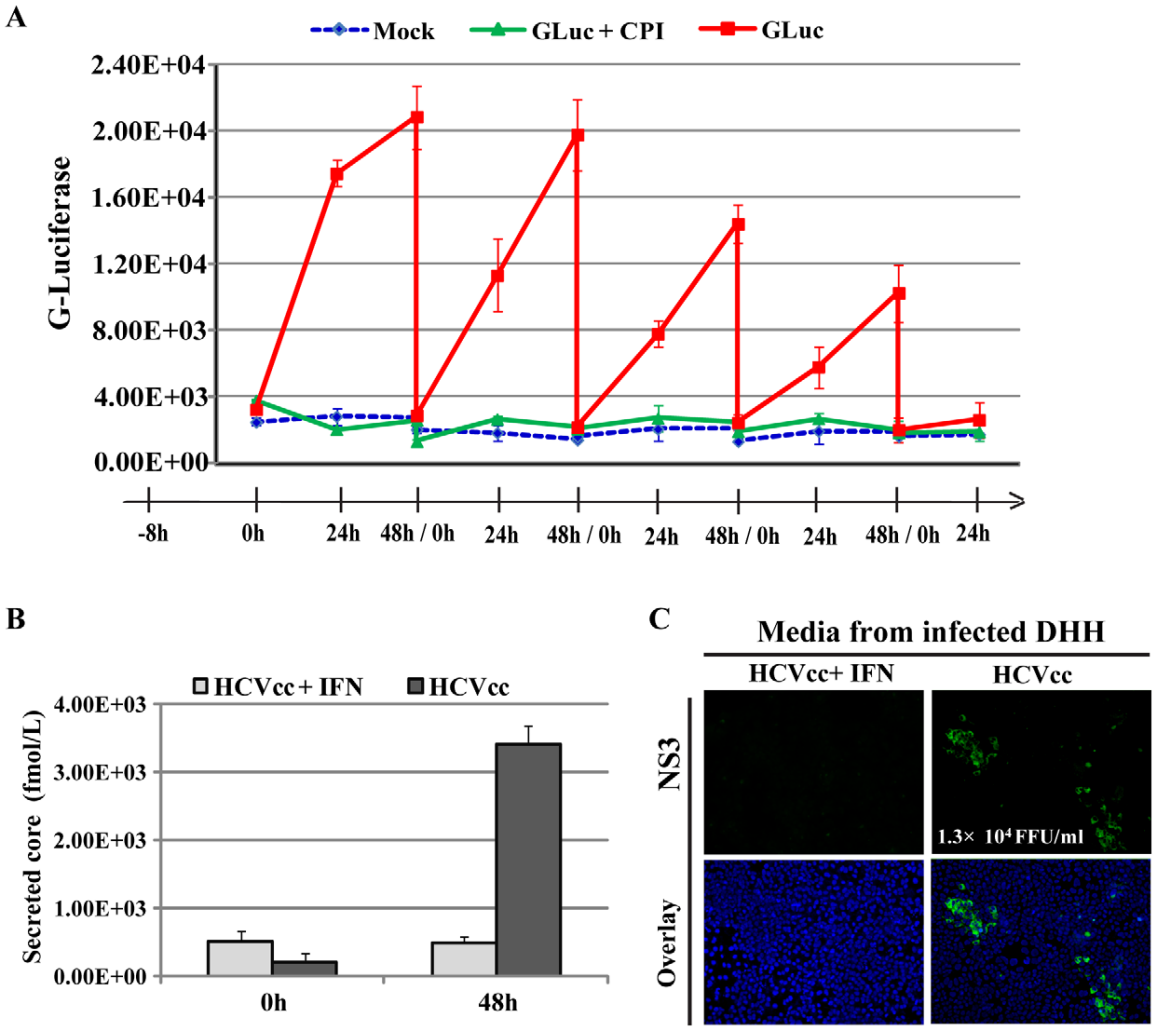
Persistent and productive infection of DHHs by HCVcc. (*A*) Continuous replication of HCVcc in DHHs. Day-10 DHHs were exposed to Jc1/GLuc2A for 9 h before the inoculum was removed and the cells were changed to medium E with or without cyclophilin inhibitor CsA at 1 µg/ml. Culture supernatants were collected daily for measurement of luciferase activity. The culture medium was replaced with thorough washing every 48 h, and CsA was included every time fresh medium was used. Error bars represent standard deviations from triplicate experiments. (*B*) Secretion of HCV core antigen into the culture medium by infected DHHs. Day-13 DHHs were exposed to HCVcc for 9 h before the inoculum was removed, and the cells washed and changed to medium E, then immediately collected as the 0-h samples. The infected cells were then incubated for an additional 48 h in medium E with or without IFN-α (50 units/ml) before the culture supernatants were collected as the 48-h samples. Error bars represent standard deviations from replicate experiments. (*C*) Reinfection of Huh-7.5 cells by HCV particles produced from DHHs. The 48-h media from (*B*) were used to infect Huh-7.5 cells, which were then fixed for NS3 staining four days after infection. The infectious titer of the HCVcc produced by DHHs is shown. FFU: focus-forming units.

To verify continuous viral replication during the infection period, we monitored the secretion of Gaussia luciferase into the culture medium by the DHHs infected with the GLuc reporter virus, using a procedure previously used to monitor persistent HCV infection in microscale PHHs [44]. After the initial infection, the viral inoculum was removed and replaced with fresh medium, a fraction of which was then collected immediately (0 h), one day (24 h), and two days (48 h) after the virus removal. At the 48-h time point, the cells were washed again and changed into fresh media which was then collected in a similar fashion. This process was repeated until day 21, when the DHHs became senescent and died off the plates. A gradual increase of the luciferase activity was detected in the culture medium after each removal, whereas the signal increase was not observed in medium from either mock infected cells or from infected cells treated with cyclosporine A (CsA), an inhibitor of cyclophilins and HCV replication [45] (Figure 3A). In addition to persistent replication, production of infectious viral particles was also achieved in DHHs infected with HCVcc. WA09-derived DHHs were infected at day 11 after differentiation, and culture supernatants were collected 48 h after infection. HCV core antigen was detected in the supernatant of the infected cells but not in that of the similarly infected but IFN-treated cells (Figure 3B). To determine whether the core-positive culture supernatant contained infectious viral particles, we used these supernatants to infect Huh-7.5 cells. NS3-positive foci could be clearly detected in the infected cells (Figure 3C), demonstrating that DHHs were capable of supporting infectious particle production.

### Transition from non-permissive to permissive cells

**Figure 4:**
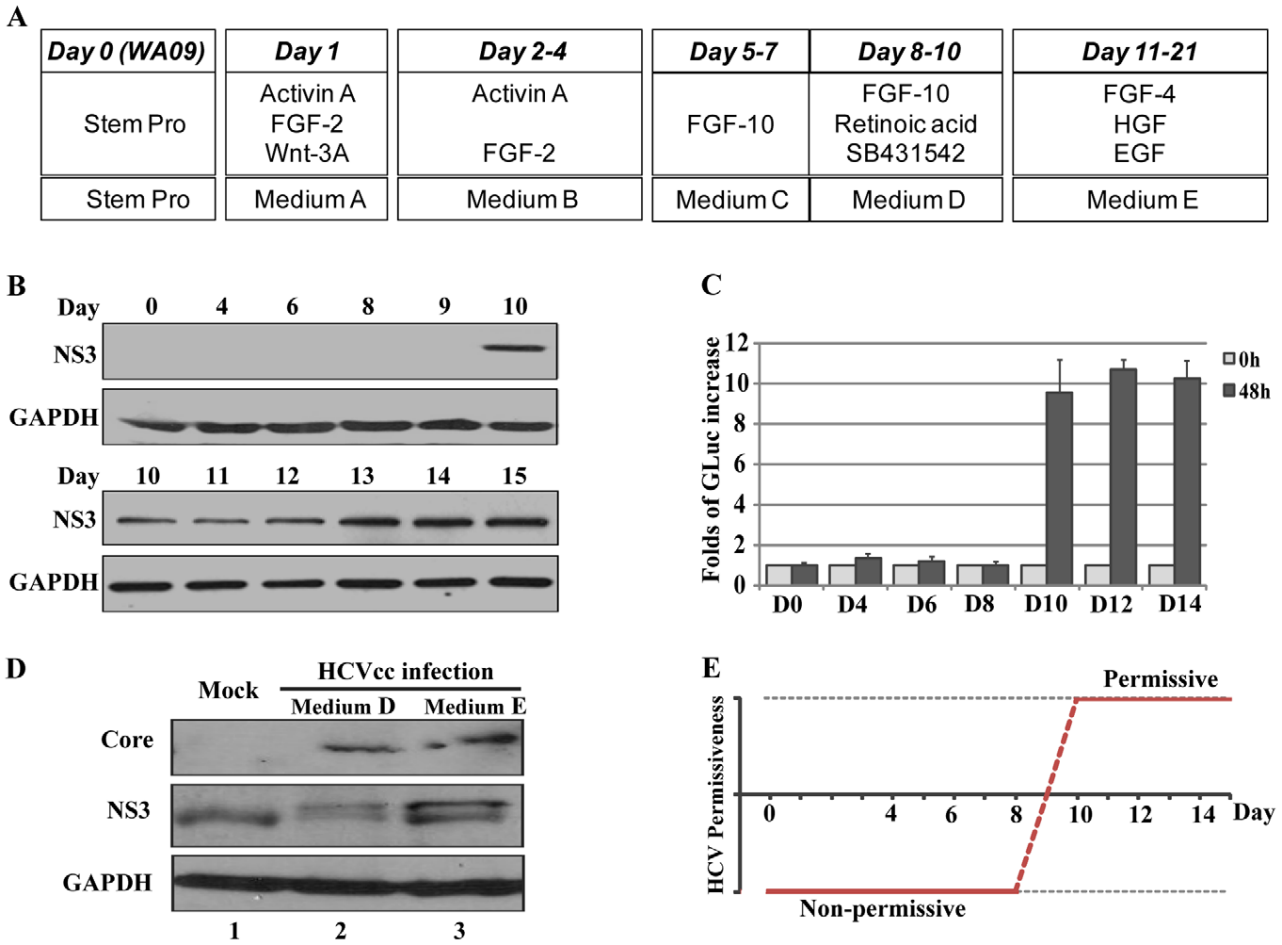
Time course of infection for determination of the transition point at which the differentiating cells became permissive for HCV. (*A*) List of growth factors in media used in the various stages of differentiation. (*B*) Time course of DHH infection. Cells were exposed for 6 h on the indicated days before the inoculum was removed. The cells were then cultured in the appropriate medium for an additional 48 h before the cell lysates were collected for detection of NS3 expression. (*C*) Secreted luciferase activities were monitored in the same experiments described in (B). Error bars represent standard deviation of triplicate experiments. (*D*) Hepatic maturation was not required for HCV infection of day-10 cells. Day-10 DHHs were infected and then either kept in medium D (hepatic specification medium) or changed to HGF-containing Medium E (hepatic maturation medium) until day 21, when all cells were collected for western blotting. The anti-NS3 antibody also recognized a nonspecific band in the mock-infected sample. (*E*) A diagram indicating the time point for transition of DHHs to HCV permissiveness on the basis of results shown in (*B*) and (*C*).

We next determined the transition stage during differentiation that rendered the DHHs susceptible to HCV infection. The hepatic differentiation protocol that we used involved five different medium compositions for the various stages of differentiation (Figure 4A). A combination of Activin A, basic fibroblast growth factor (b-FGF), and Wnt-3A (Media A and B) was used to induce the differentiation of definitive endoderm (days 1–4), which was cultured in a FGF-10-containing medium (medium C) for three days (days 5–7) for initiation of definitive endoderm hepatic specification. After day 7, medium C was supplemented with retinoic acid (RA) and a transforming growth-factor-β (TGF-β) inhibitor, SB431542, and the cells were cultured for three additional days (days 8–10) in this medium (medium D). Finally, the hepatocyte-like cells were allowed to mature in medium E, which contained hepatocyte growth factor (HGF), epidermal growth factor (EGF), and FGF-4 (days 11–21). We exposed cells at different time points to GLuc-based HCVcc for 6 h, removed the inoculum, and then monitored infection by measuring both intracellular NS3 expression and luciferase activity in the medium 48 h after infection. A clear infection signal was detected in cells at and after day 10 after differentiation, whereas the stem cells (H9), the definitive endoderm, and cells up to day 9 after differentiation could not be infected (Figure 4B and 4C). Because the day-10 cells were normally changed into medium E immediately after the removal of the viral input, we wanted to determine whether medium E was required for the infection. To address this question, we performed an experiment in which the infected day-10 cells were either kept in medium D (FGF-10, RA, and SB) or changed into medium E (HGF, EGF, and FGF-4). Both samples were collected at day 21 and subjected to immunoblotting for detection of HCV proteins. medium E was not required for HCV permissiveness, as both cell populations became infected, but the maturation process may further increase the infection efficiency (Figure 4D, compare lanes 2 and 3). These results identify a discrete temporal switch during the hepatic differentiation process that marks the transition to permissiveness for HCV infection (Figure 4E).

### Cellular changes associated with HCV permissiveness

**Figure 5:**
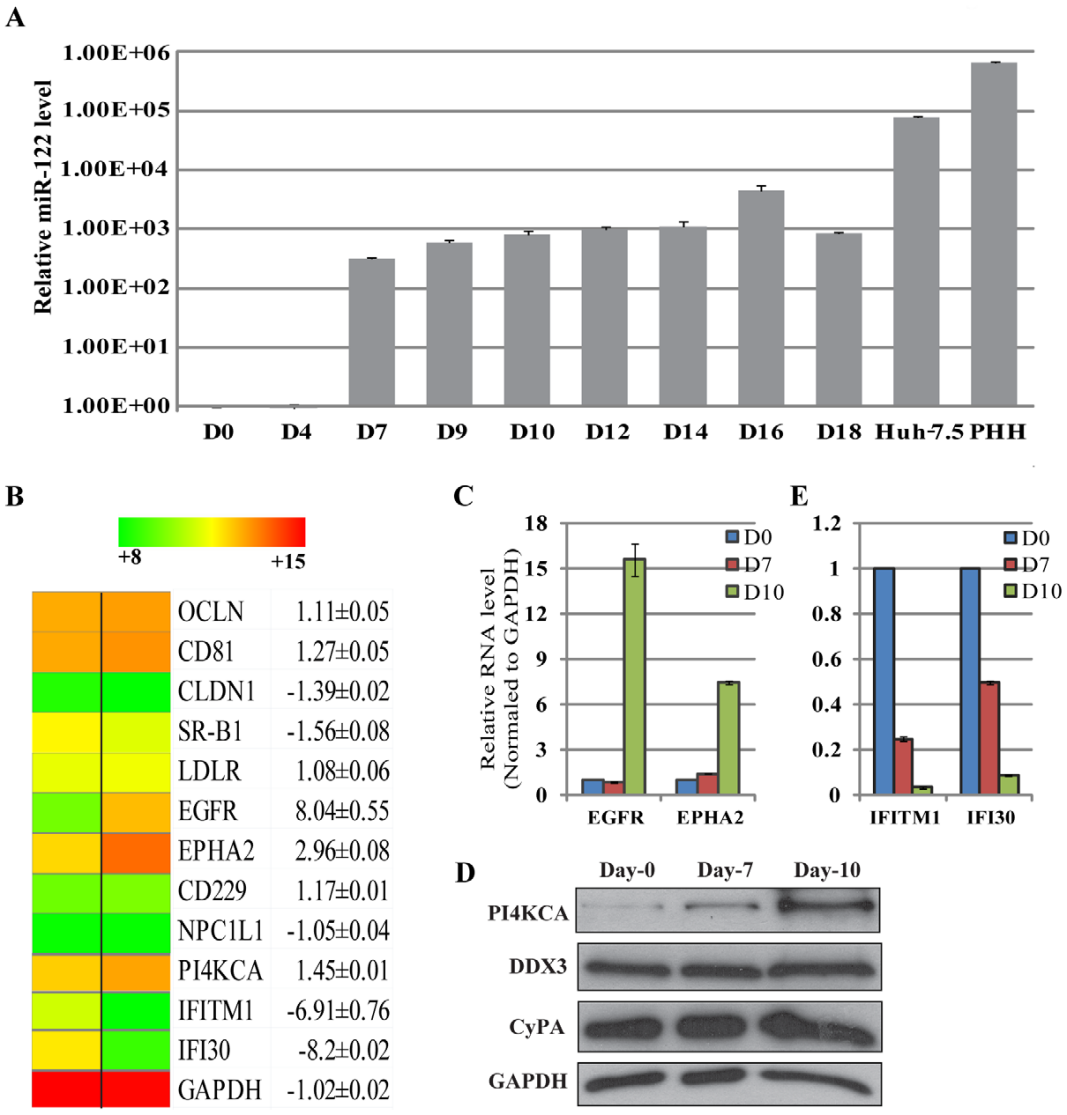
Cellular determinants of HCV susceptibility. (*A*) Induction of microRNA miR-122 expression by FGF-10 during hepatic specification. Equal amounts of total cellular RNA from various cells at the indicated days were subjected to a real-time RT-PCR assay for detection of miR-122 expression. (*B*) Microarray heat map of gene expression levels in day-10 versus day-7 cells. Two independent RNA samples were processed for each time point. The numbers represent the average values and standard deviations. The conventional color spectrum with green representing downregulation and red representing upregulation was adopted. Fold of changes were also listed next to the name of the gene. (*C*) Quantitative RT-PCR results of EGFR and EphA2 induction. (*D*) Upregulation of PI4KIIIα protein during the differentiation process. The levels of CyPA and DDX-3 remained unchanged in the same samples. (*E*) Quantitative RT-PCR results of IFITM1 and IFI30 expression induction.

We then sought to identify the cellular determinants whose induction or repression by the hepatic specification process was correlated with permissiveness to infection. Liver-specific genes that are important for HCV infection are good candidates for such determinants. The microRNA miR-122 is such a cellular cofactor [46]–[48]. Expression of miR-122 was not detectable by real-time RT-PCR in day-0 or day-4 cells but was greatly induced at day 7 and then maintained throughout the differentiation process (Figure 5A). These data suggested that the induction of miR-122 expression by hepatic specification conditions contributed to, but was not sufficient for, the transition from non-permissiveness to permissiveness. We next performed microarray analysis to compare gene-expression profiles of day-7 (non-permissive) and day-10 (permissive) cells. The addition of medium D resulted in changes in expression levels of hundreds of genes, many of which are associated with cell signaling pathways or function of extracellular components (Dataset S1). We focused on genes that have been previously implicated in HCV infection. Expression of the four well-characterized receptors (cluster of differentiation 81 (CD81), SR-BI, claudin-1, and occludin) remained largely unchanged, as did the expression of the putative attachment factor, the low-density lipoprotein receptor (LDL-R, Figure 5B). The expression of epidermal growth factor receptor (EGFR) and ephrin receptor A2 (EphA2), two receptor tyrosine kinases (RTKs) identified in an siRNA library screening for HCV entry factors [49], increased in day-10 cells (Figure 5B). Quantitative RT-PCR confirmed the upregulation of these genes (Figure 5C) to be comparable with the levels found in PHHs (Figure S2A in Text S1). In addition, phosphatidylinositol 4-kinase type III alpha (PI4KIIIα), another critical HCV cofactor [50]–[52], was also induced in day-10 cells, especially at the protein level (Figure 5D). In contrast, the expression of most other reported cellular cofactors of HCV remained unchanged (Figure S2 in Text S1). Immunostaining of cell surface receptors confirmed the RNA data from microarray and conventional RT-PCR (Figure S3 in Text S1). Finally, there were also many genes that were down-regulated in day-10 cells compared to day-7 cells. One of these encoded the interferon-induced transmembrane protein 1 (IFITM1) (Figure 5E), an interferon-stimulated gene (ISG) recently shown to repress HCV replication and down-regulation of which by siRNA increased HCVcc infection in Huh-7.5 cells [53]. Taken together, these results suggest that transition to HCV permissiveness during the in vitro differentiation process may require both the activation of positive factors (miR122, EGFR/EphA2, PI4KIIIα etc.) and the downregulation of antiviral genes such as IFITM1.

### Genetic modification to generate HCV-resistant DHHs

**Figure 6:**
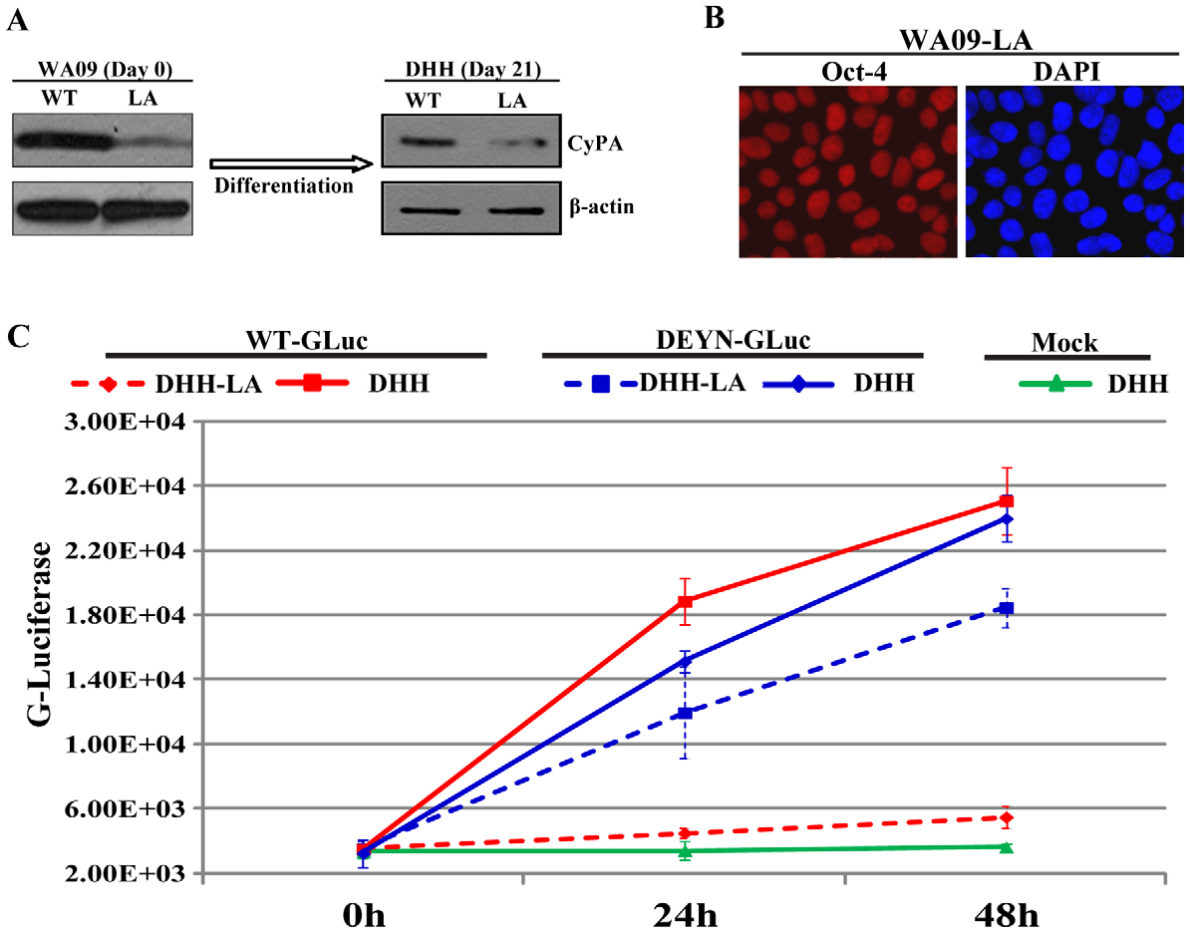
Genetic modification of hESCs and HCV-resistant DHHs. (*A*) Suppression of CyPA expression by shRNA in WA09 cells and day-21 DHHs. (*B*) CyPA knockdown did not affect the expression of pluripotency marker Oct-4 in WA09 cells. (*C*) Modified DHHs were resistant to wildtype HCV infection. Infection of both the wildtype and CyPA-KD (LA) DHHs were done at day 13 and allowed to proceed for 48 h. Luciferase in the culture supernatant for monitored. Wildtype HCVcc (Jc1/GLuc2A) infected unmodified DHHs but not CyPA-KD DHHs (redlines), and the DEYN mutant infected both cell types (blue lines). Error bars represent standard deviations of replicate experiments.

A distinct advantage of DHHs over PHHs is the potential to modify the cells genetically at the pluripotent stage and then produce DHHs with the desired phenotype. We introduced a small-hairpin RNA (shRNA) directed at cyclophilin A (CyPA) into WA09 cells by lentiviral vector-mediated gene delivery. This shRNA, sh-A161, had previously been shown to block HCV infection in a human hepatoma cell line Huh-7.5 by knocking down expression of CyPA [37]. The importance of CyPA in the HCV life cycle has been validated clinically with cyclophilin inhibitors in patient trials [54]. Suppression of CyPA expression in WA09 cells was similarly achieved by stable expression of sh-A161 (Figure 6A), and the resulting KD cell line (WA09-LA) retained normal expression of the pluripotent marker Oct-4 (Figure 6B). When WA09-LA cells were subjected to the hepatic differentiation procedure to produce DHH-LA, the knockdown of CyPA was maintained in the differentiated cells (Figure 6A), indicating long-term suppression of gene expression by shRNA was not affected by the differentiation steps as long as a house-keeping promoter was selected to drive the shRNA expression (e.g. a murine U6 promoter contained in the lentiviral construct used in this study). Infection by wildtype HCVcc, however, was reduced to the mock level in DHH-LA cells (Figure 6C, red dotted lines) cells. Importantly, these cells remained permissive to infection by a CyPA-independent mutant virus (GLuc-DEYN) (Figure 6C, blue lines), recently isolated by means of a genetic approach termed cofactor-independent mutant (CoFIM) selection [55]. These data suggest that the block to HCV infection was due to CyPA knockdown rather than to a non-specific effect of the shRNA expression [56]. A second WA09 line harboring an shRNA directed at PI4KIIIα also produced HCV-resistant DHHs upon differentiation (Figure S4 in Text S1), lending further support to the broad utility of the modification/differentiation technology.

### Patient serum–derived HCV infects DHHs but not Huh-7.5 cells

**Figure 7:**
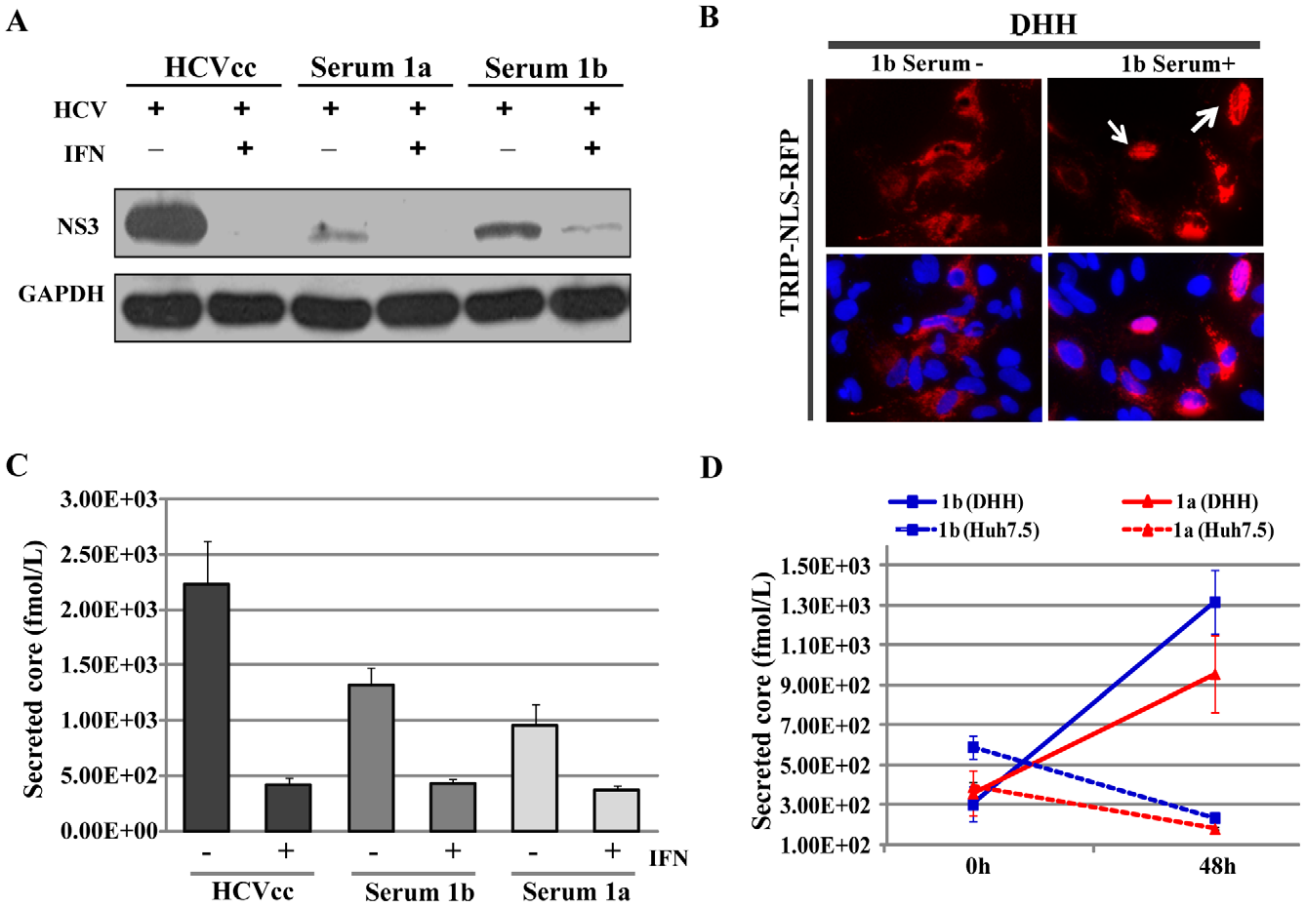
Direct infection of DHHs by HCV derived from patient serum. (*A*) Detection by western blotting of IFN-sensitive infection by HCV particles derived from patient serum (HCVser). IFN-α was included in the medium at 50 units/ml when indicated. The multiplicity of infection for the individual viruses was: HCVcc: 0.5; Serum 1a: 0.02; Serum 1b: 0.5. (*B*) Visualization of single-cell infection events by HCVser with the HCV-dependent fluorescence relocalization assay. Arrows indicate individual cells infected with genotype 1b HCVser and showing nuclear translocation of the RFP. (*C*) Secretion of HCV core antigen into culture supernatant by HCVser-infected DHHs. Values for core levels in supernatants collected 48 h after infection were plotted. IFN-α was included in the medium at 50 units/ml when indicated. Error bars represent standard deviation of replicate experiments. (*D*) HCVser preferentially infected DHHs over Huh-7.5 cells. Equal amounts of genome equivalent of HCVser were used to infect either Huh-7.5 or day-11 DHHs. Core levels in the supernatants collected at 0 and 48 h were plotted for both cell lines. Error bars represent standard deviations of replicate experiments.

Although robust infection of PHHs by HCVcc has been reported [16], [44], [57], direct infection by HCVser remained inefficient [16], [58]. We infected DHHs with HCVser of two genotypes: a genotype-1b patient serum that contained high-titer HCV RNA copy numbers (1.8×10^6^ copies/ml) and a genotype-1a patient serum that had been previously demonstrated to be infectious in the *Alb-uPA* mouse model [5] (RNA titer of 1×10^6^ copies/ml). The DHHs were infected at the indicated multiplicity of infection for 48 h before the cells were lysed for analysis of HCV protein expression. Infection was readily detectable by western blotting and sensitive to IFN inhibition (Figure 7A), although the infection signal of HCVser was weaker than that of the HCVcc. HCVser infection was also detectable with the HDFR assay (Figure 7B). In addition, secretion of HCV core antigen was detected in the supernatant of the DHHs infected by HCVser (Figure 7C). In contrast, exposure of Huh-7.5 cells to HCVser of a multiplicity of infection up to 0.5 did not result in detectable intracellular expression of NS3 (data not shown) or any release of HCV core into the culture medium (Figure 7D). Genome sequencing did not reveal any adaptive mutations that have been reported in the literature (data not shown). Given the high permissiveness of Huh-7.5 cells to HCVcc infection, these results strongly suggest that HCVser preferentially infects the non-cancerous DHHs.

## Discussion

We have demonstrated that hepatic cells derived by directed differentiation of stem cells, including iPSCs, can support HCV infection. Complete life cycles of HCV infection could be completed starting with HCV entry and ending with secretion of infectious viral particles into culture media. Infection of DHHs was sensitive to replication inhibitors as well as entry blockers. Four different variants of JFH-1, including a J6/JFH hybrid (GLuc), were used to produce HCVcc used in this study. Both wild-type sequence (JFH-FLAG) and adaptive mutants (SAV and Mut4-6) were able to replicate in DHHs, indicating that the ability for DHHs to support HCV infection did not depend on particular isoforms or mutations. In addition, successful infection by two clinical isolates of genotype 1a and 1b demonstrated the feasibility of using DHHs to study these genotypes that are prevalent in patients but understudied in cell culture [25], [59]. Beyond the genotype considerations, direct infection by patient serum also has broad significance for challenging research areas such as the dissection of drug resistance mechanisms and functional characterization of authentic HCV particles. Silberstein et al. [60] recently demonstrated the long-term passage of a genotype 1b clinical isolate in a monkey kidney cell line (VeroE6) that was defective in type I IFN production. High titer of infectious viruses could be recovered and this isolate was able to recapitulate the in vivo IFN resistant phenotype in cell culture. This virus, however, was highly cytotoxic to Huh-7.5 cells, somewhat limiting the study of persistent infection in human hepatic cells. Interestingly, although the 1a serum used in this study was obtained from a patient who was discontinued from pegylated IFN/Ribavirin with significant side effects and poor response to treatment, infection by this virus was sensitive to IFN treatment in vitro. Host determinants may have been responsible for the IFN resistance observed in vivo for this patient.

The DHHs represent an important addition to the small field of in vitro models for HCV infection. In contrast to the cell lines derived from tumor tissues, DHHs are non-cancerous and retain important functions of primary hepatocytes such as secretion of ALB, glycogen storage, LDL uptake, cytochrome P450 function, and the ability to replace mouse hepatocytes in liver injury mouse models. DHHs also offer advantages over PHHs as being more accessible, genetically malleable, and unlimited in supply. Several groups recently reported the direct induction of mouse fibroblasts into hepatocyte-like cells (iHep) [61], [62]. Whether a similar feat can be accomplished for human cells and, if so, whether the iHep cells will have enough proliferative potential to serve as a useful model for HCV research remain to be determined.

Genetic modification of pluripotent stem cells before directed differentiation is an attractive approach to obtaining specific cell types with desired phenotypes. In the context of HCV infection and liver disease, stem-cell lines with essential cellular cofactors knocked out or knocked down can serve as a renewable source of HCV-resistant hepatocyte-like cells in vitro, which can in turn be used in transplant experiments. Even though most cellular proteins probably play essential roles normally, and their silencing cannot be reasonably expected not to affect the host, the opportunity for inhibiting a cellular cofactor does sometimes arise as a result of functional redundancy at the cellular but not the viral level, as is the case with the HIV coreceptor C-C chemokine receptor type 5 (CCR5) [63]. For RNA viruses with high mutation and turnover rates, inhibiting a cellular rather than a viral target may offer the advantage of a higher genetic barrier to development of resistance.

Gene knockout technology in mouse embryonic stem cells revolutionized the field [64] and remains the gold standard for definitive studies of gene function, but the robustness of the technology did not transfer to hESCs easily [65]. The efficiency of homologous recombination in hESCs and human iPSCs is much lower, in part because the pluripotent state of the human cells resembles that of the mouse-derived epiblast stem cells, rather than the true naïve state of the mouse embryonic stem cells [66], [67]. Further reprogramming of human iPSCs with leukemia inhibitory factor [67] or significantly increasing the size of the targeting vector [68] may be required to produce an acceptable rate of recombination. RNA interference, on the other hand, appears to function efficiently in all cell types and represents an alternative to gene knockout, especially when partial suppression of a cofactor is sufficient to reduce viral infection in a meaningful way. This study demonstrated that lentiviral vector–mediated expression of shRNA can be maintained in long-term differentiation cultures and that CyPA KD in hESCs or DHHs has no apparent adverse effects on pluripotency or differentiation. The CyPA KD DHHs were permissive to infection by an HCV mutant with reduced dependence, further indicating that these modified cells retained hepatic features that encompass HCV's liver tropism. These data are also consistent with the finding that CyPA-null mice developed normally and had life expectancy comparable to that of wildtype mice [69].

The efficiency of PHHs to support HCV production is typically much lower than that of Huh-7.5 cells and varies in different studies [16], [44], [57], presumably because of the different batches of PHHs used or cell culture conditions or both. A similar situation was observed with DHHs: despite robust intracellular expression of HCV proteins and unequivocal evidence of virion production in the culture medium of the infected DHHs, the infectious titers have so far remained relatively low. This could be due to interferon produced in the medium or may reflect the inherently low infection efficiency in primary cells [17], [70]. In addition, expression of liver-specific marker genes such as ALB is much lower in DHHs than in PHHs, suggesting the differentiation protocol could be further optimized. Our preliminary experiments showed that DHHs cultured in three-dimensional cell-culture scaffolds conferred higher infectivity to HCVcc (Figure S5 in Text S1), pointing to the possibility of improving DHH infection efficiency by means of tissue engineering, as has been reported for PHHs [44]. Interestingly, the relative efficiencies with which Huh-7.5 and DHHs support HCVcc and HCVser infections were distinctly different. HCVcc infected DHHs less efficiently than they did Huh-7.5 cells, whereas HCVser specifically infected DHHs but not Huh-7.5, suggesting that DHHs represent a more physiologically relevant model for infection by clinical isolates of HCV. Similarly, a GT1a infectious clone that failed to replicate in Huh-7.5 cells was able to replicate and produce low numbers of viral particles in PHHs cocultured with hepatic stellate cells [57]. The mechanism underlying this interesting phenomenon is unclear at the present time, but may be related to, among other possibilities, the different genotypes represented by HCVser and HCVcc used in these studies. Both the Banaudha [57] study and ours used HCVser of genotypes 1a and 1b whereas the HCVcc were based on JFH-1 or J6/JFH, both of genotype 2a. It has been documented that HCVcc based on genotype 1a is significantly less infectious than the JFH-1-based HCVcc in Huh-7.5 cells [25].

Viral tropism for a specific cell type is typically associated with the expression of tissue-specific cofactors (e.g. receptors). HCV infection is largely hepatotropic although the virus has been reported to infect other cell types, including B-cell lymphoma cells [71]. Viral entry into DHHs by HIV and VSV particles pseudotyped with HCV envelope proteins has been reported [35], [72], consistent with our finding that all known HCV receptors are expressed on DHHs. We also found that the induction of miR-122 expression was correlated with hepatic specification and preceded the transition to HCV susceptibility, confirming the connection between this liver-specific microRNA and host restriction in non-hepatic cells, as first reported by Joplin et al [47]. FGF-10 treatment, possibly in combination with the withdrawal of Activin A, increased miR-122 expression by more than several hundred fold. The link between FGF-10 and miR-122 induction may be the hepatocyte nuclear factor 4 alpha (HNF4α) which, along with HNF6α, has recently been reported to bind the miR-122 promoter and activate pri-miR-122 transcription [73], [74]. The expression HNF4α itself may be regulated by FGF-10 as mutant zebrafish lacking the fgf-10 gene showed misexpression of HNF4α [75]. FGF-10 has also been shown to be crucial for hepatoblast survival and proliferation [76], and an important role of miR-122 in hepatic development has been demonstrated in zebrafish [73], perhaps not surprising for a molecule that is highly liver specific and extremely abundant (over 50,000 copies per cell in mouse liver versus less than 50 copies in other tissues) [77]. It is thus tempting to speculate that FGF-10 in part exerts its effect on liver growth via the actions of miR-122. Besides miR-122, EGFR and EphA2, two RTKs that contribute to the HCV entry process through their kinase function, were specifically upregulated in permissive cells. Of note, medium E, which contains EGF, increased HCVcc infection of day-10 cells, consistent with previously reported results in Huh-7.5 and PHHs [49]. The expression of both ephrin A1, which is the ligand for EphA2, and ephrin B2 also increased from day 7 to day 10. The latter is the membrane-bound ligand for EphB and serves as a cellular receptor for Nipah virus [78]. Whether it also plays any role in the HCV entry process remains to be determined.

To our knowledge, ours is the first report of any cell type that can be rendered permissive to HCV infection and replication by treatment with defined chemical compounds. This important advance opens up new possibilities for identifying novel signaling pathways required for viral infection and could lead to the discovery of new drug targets for HCV. Moreover, we have shown that pluripotent stem cells can be genetically modified before differentiation and then generate virus-resistant hepatocytes. In addition to direct applications in studies of cellular cofactors in infections or other diseases with a genetic component, the concept illustrated here can be coupled with patient-specific iPSC technology, especially if the potential immunogenicity issue [79] can be overcome, to generate a multitude of cell types with desired phenotypes for cell therapy.

## Materials and Methods

### Growth factors, chemicals and antibodies

Basic FGF (b-FGF), Stem Pro hESC SFM, β-mercaptoethanol, and Geltrex were purchased from Invitrogen (Carlsbad, CA); FGF-10, FGF-4, EGF, and HGF from PeproTech (Rocky Hill, NJ); SB 431542 and retinoic acid from Sigma Aldrich (St Louis, MO); Wnt-3A from Stemgent (San Diego, CA); Accutase from Innovative Cell Technologies (San Diego, CA); Activin-A from R&D systems (Minneapolis, MN); and Probumin from Millipore (Billerica, MA). A list of antibodies used, along with providers and catalog numbers, is given in Table S1 in supporting information S3.

### hESC, iPSC, and primary human hepatocytes

Human ESC line WA09 (H9) and iPS line iPS.K3 cells were obtained from WiCell Research Institute and Stephen Duncan at Medical College of Wisconsin, respectively. Stem cells were maintained on Geltrex coated culture plates in Stem Pro medium (Invitrogen, Carlsbad, CA). Freshly isolated PHHs were purchased from Celsis In Vitro Technologies (Baltimore, MD) and maintained according to provider's instructions.

### Differentiation of hESCs and iPS.K3 into hepatic cells

The base defined medium (DM) consisted of DMEM/F12 containing 10% Probumin, 0.2% β-Mercaptoethanol, 1% L-Alanyl-L-Glutamine and 2% hESC supplement. Confluent cells were harvested with Accutase and then plated into culture dishes (Costar; Corning Life Sciences) precoated with Geltrex (1∶200 dilution in DMEM/F-12) in Stem Pro medium at a confluence level of 30–40%. The next day, culture medium was changed to medium A (DM+100 ng/ml Activin-A+8 ng/ml b-FGF+25 ng/ml Wnt-3A) for 24 hrs, followed by three days in medium B (DM+100 ng/ml Activin-A+8 ng/ml b-FGF). To induce hepatic differentiation, we then cultured cells in the presence of medium C (DM+50 ng/ml FGF-10) for three days and then in the presence of medium D (DM+50 ng/ml FGF-10+0.1 µM RA+1 µM SB431542) for three more days. The immature hepatocyte-like cells were then split 1∶2 and grown in medium E (DM+30 ng/ml FGF-4+50 ng/ml EGF+50 ng/ml HGF) for 10 days with changes to fresh medium E every two to three days.

### Periodic acid-Schiff staining

The PAS staining was done on the stem cells, the day-18 DHHs, and freshly isolated PHHs using a commercial kit (Sigma-Aldrich, St. Louis, MO) per instructions provided by the manufacturer.

### HCVcc and HCVser used in the infections

All JFH-1 based HCVcc (Mut4-6, SAV, Jc1/GLuc2A, and DEYN-Jc1/GLuc2A) were produced in Huh-7.5 cells as previously described [20]. The genotype 1b HCV serum was obtained from a commercial supplier (Teragenix, Ft. Lauderdale, FL), and the 1a serum has been previously described [5]. All infections were performed by incubation of virus inoculum with cells for 6–9 h before the cells were washed and changed into the medium appropriate for the specific cell type and differentiation stage. For the time course of DHHs permissiveness, infection at each time point was allowed to proceed for exactly 48 h before cell harvesting and western blotting. Viral titers of HCVcc produced from DHHs were performed with Huh-7.5-based cells and measured in focus-forming units (FFU) per milliliter.

### Immunofluorescence analysis of HCV receptors and intracellular antigens

Cells were fixed in 4% paraformaldehyde in phosphate-buffered saline (PBS) at room temperature for 10 min and blocked with PBGB (PBS containing 10% normal goat serum, and 1% bovine serum albumin (BSA)) at room temperature for 2 h. Cells were incubated with primary antibodies (anti-CD81, anti-SR-B1, anti-claudin 1 and anti-occludin, diluted in PBG at 1∶200) at 4°C overnight or 2 hrs at room temperature. Isotype mouse or rabbit IgGs were used as negative controls. After four washes with PBSB (PBS with 0.1% BSA), FITC or TRITC-conjugated secondary antibody diluted at 1∶500 was added and incubated at room temperature for 1 hr. Before being mounted with VECTASHIELD (H-1200, Vector Labs), cells were washed with PBSB three times and once with PBTG (PBS containing 0.1% Triton X-100, 10% normal goat serum, and 1% BSA). For intracellular staining, we permeabilized the cells in PBST after fixing to allow access by primary antibody.

### HCV-dependent fluorescence relocalization assay

Lentiviral vectors expressing EGFP-IPS (TRIP-EGFP) or RFP-NLS-IPS (TRIP-NLS-RFP) were provided by Charles Rice and produced in 293-FT cells as previously described [41]. Day-10 DHHs or Huh-7.5 cells, seeded on coverslips the day before, were transduced with the vectors for 24 h before being exposed to HCVcc or HCVser for 6 h. The cells were cultured for 2–3 more days before the slides were fixed for fluorescence microscopy analysis.

### Microarray and RT-PCR analysis

Complimentary DNA used for microarray hybridization was prepared as follows. Total RNAs from day-7 and day-10 cells were isolated with the Qiagen RNeasy Mini kit, and RNA was converted into single-stranded cDNA with the High Capacity cDNA Reverse Transcription kit (Applied Biosystems). The RNA/cDNA hybrids were denatured at 95°C for 1 min and then treated with RNase A for 30 min at 37°C. The resulting cDNA was cleaned up with the Qiagen PCR purification kit before being used for fluorescent labeling. A Nimblegen 4×72K Expression Array was used for hybridization according to the manufacturer's instructions. Expression data and gene ontology analysis were done with ArrayStar (DNASTAR) and Gorilla (Technion - Laboratory of Computational Biology). For RT-PCR, total RNA was isolated from various days post-differentiation using TRIzol and then converted to first-strand cDNA with SuperScript III (Invitrogen) with oligo-dT serving as the RT primer. The resulting products served as templates for PCR analysis of HCV cofactors and receptors. Primer sequences for the hepatic markers and HCV cellular cofactors are available upon request.

### Real-time RT-PCR detection of micro-RNA 122 (miR-122)

To determine miR-122 levels, we reverse transcribed TRIzol-extracted RNA samples using the TaqMan MicroRNA Reverse Transcription kit (Applied Biosystems), and the resulting cDNA served as templates for real-time PCR analysis with the TaqMan MicroRNA Assay for miR-122 (Applied Biosystems).

### Albumin and HCV Core ELISA

Albumin ELISA was performed with a human Albumin ELISA kit (Bethyl Laboratories, Montgomery, TX), and HCV core ELISA with the HCV Antigen ELISA kit (Ortho-Clinical Diagnostics, Japan), according to manufacturer's instructions.

### Lentivirus-mediated RNA interference

Lentiviral vectors containing a shRNA directed at human CyPA has been described previously [37]. A shRNA directed at PI4KIIIα was constructed in a similar fashion. The shRNA target sequence of the PI4KIIIα mRNA is 5′-AAG CTA AGC CTC GGT TAC AGA-3′. These vectors were introduced into stem cells by the standard lentiviral transduction procedure [80], and stable cells harboring shRNA were selected by culture of the cells in Stem Pro medium supplemented with 600 ng/ml of puromycin.

## Supporting Information

Dataset S1 **Microarray data comparing gene expression profiles of day-7 and day-10 cells.** List of genes and their changes (folds up- or down- regulated) from day-7 to day-10 are shown for two independent microarray experiments.
(XLSX)

Text S1 **Supplemental** **Figures 1** **–** **5** **.** **Figure S1.** Expression of mRNAs of hepatic markers during differentiation. Expression levels were normalized to GAPDH and the those of PHH was set to be 1. ASGR1: asialoglycoprotein receptor-1; CyP3A: cytochrome P450, family 3, subfamily A; PHH: primary human hepatocytes. **Figure S2.** Expression of mRNAs of HCV cofactors during hepatic differentiation. (*A*) Expression of EGFR and EphA2 mRNA in DHHs and PHHs. (*B*) Microarray heat map of expression levels of reported HCV cofactors in day-10 and day-7 cells. (*C*) Expression profile of HCV cofactors as represented by conventional RT-PCR and gel analysis. **Figure S3.** HCV receptor molecules expressed in stem cells. (*A*) Cell-surface staining of the four well-characterized receptors (CD81, SR-BI, Claudin-1, and Occludin) for HCV entry in both H9 and day-10 cells. (*B*) RT-PCR analysis of receptor expression during the hepatic differentiation process. **Figure S4.** PI4KIII knockdown in DHHs block HCV infection. (A) Suppression of PI4KIIIα by shRNA in Huh-7.5 cells. (B) PI4KIIIα KD efficiently blocked HCV infection in Huh-7.5 cells. (C) PI4KIIIα KD in H9 cells. (D) DHHs with PI4KIIIα KD were resistant to HCV infection. The cells were infected at day 13, and the luciferase activity was monitored for the next 48 h. Error bars represent standard deviations of replicate experiments. **Figure S5.** Increased infection efficiency of DHHs cultured in three-dimensional scaffolds. For the 3-D cultures, day-9 cells were seeded onto either polystyrene or polycaprolactone scaffolds, which were transferred to a new dish after adherence of the cells. Infections by Jc1/GLuc2A were performed at day 13, and luciferase assays in the next two days. The luciferase results were normalized to the cell numbers and then compared with those of the regular (2-D) cultures, which were set to be 100%. Error bars represent standard deviations of replicate experiments.
(PDF)

Table S1 **Antibody list.** Name and source of the antibodies (suppliers and catalog numbers) used in this study are listed.
(DOC)
