# Supplementary figures and images for: Productive Hepatitis C Virus Infection of Stem Cell-Derived Hepatocytes Reveals a Critical Transition to Viral Permissiveness during Differentiation
Source: PLoS Pathog. 2012 Apr 5;8(4):e1002617. doi: 10.1371/journal.ppat.1002617 (PMC3320597; doi:10.1371/journal.ppat.1002617)

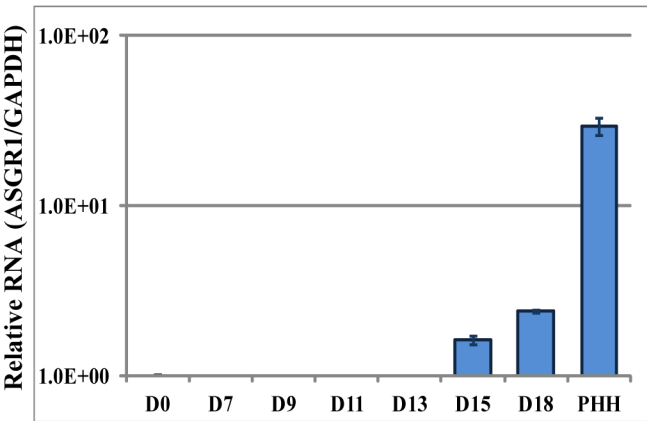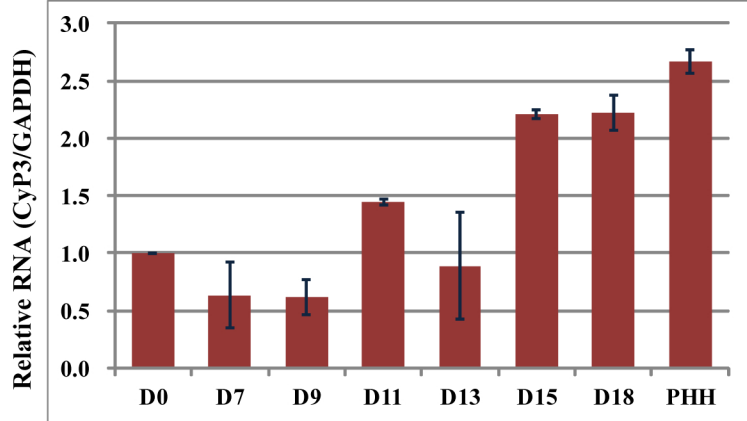

A

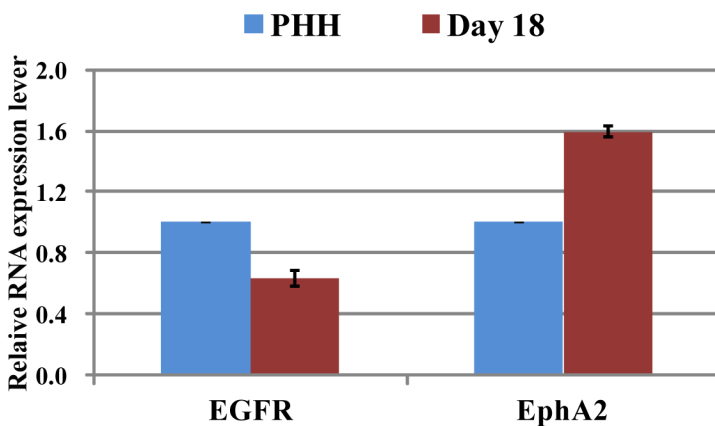

B

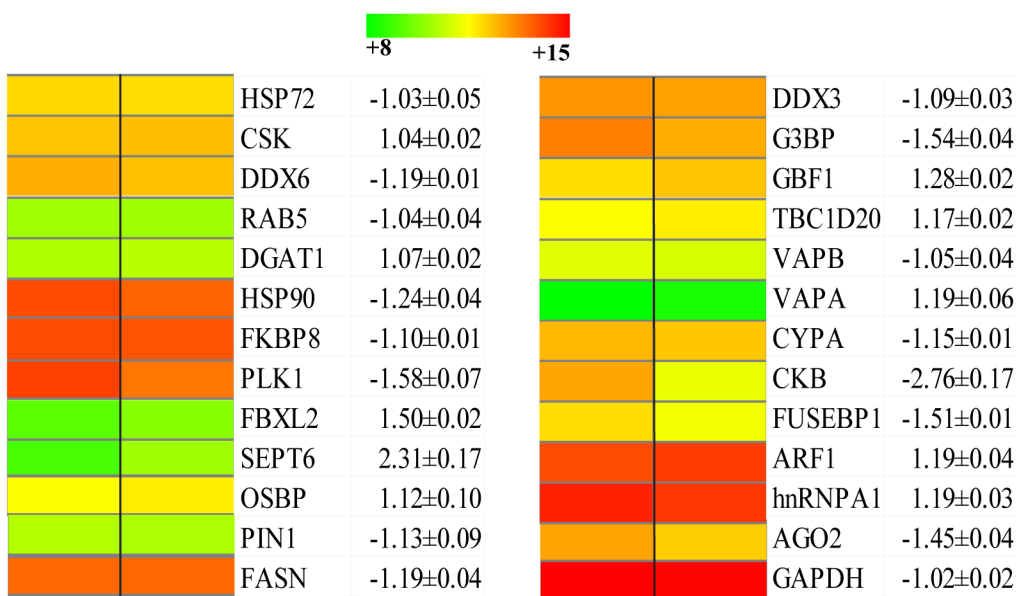

C

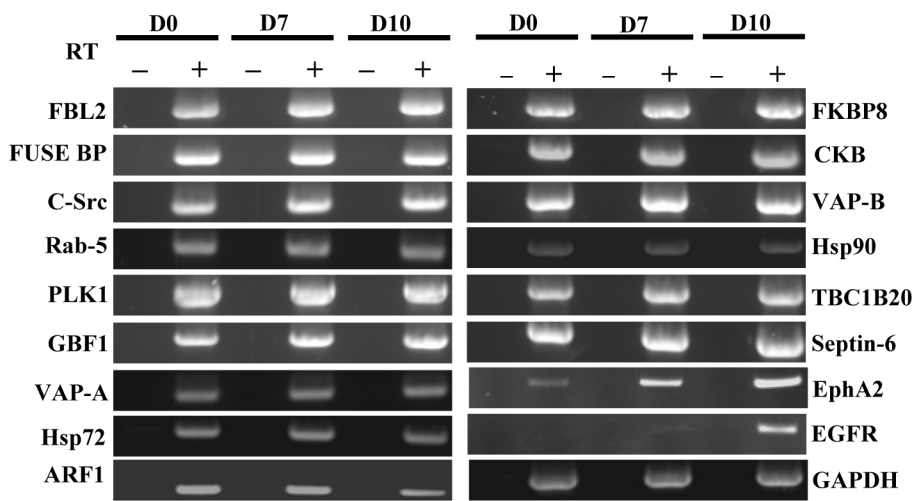

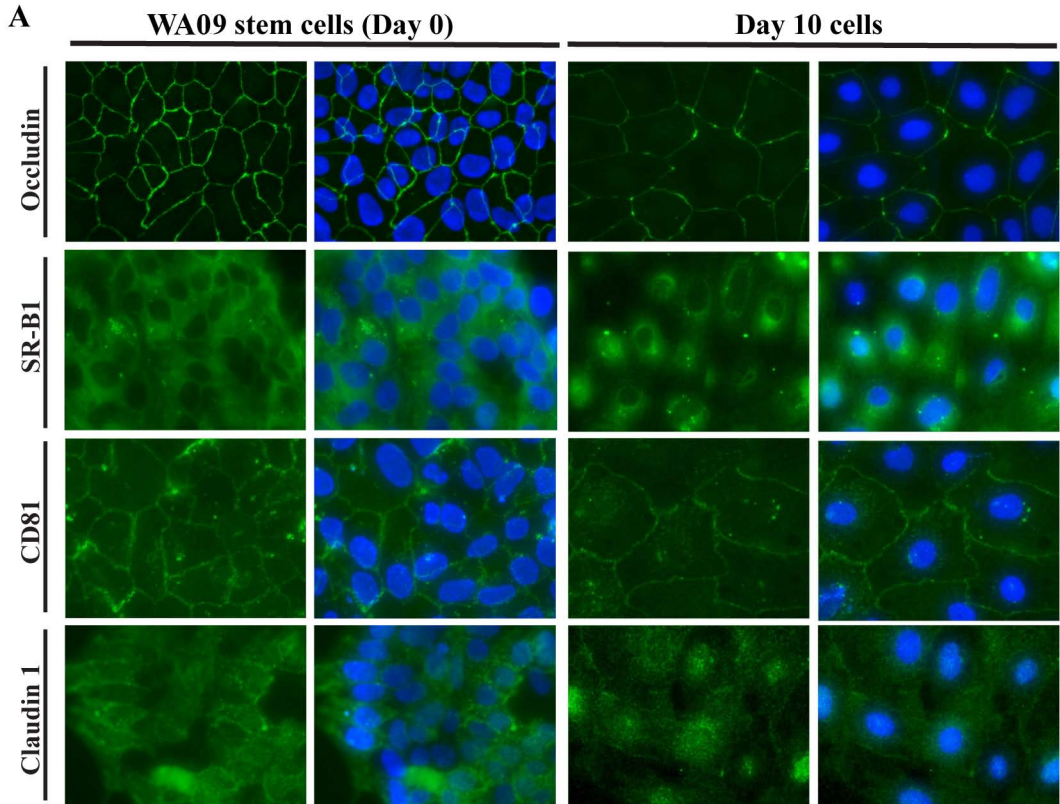

**B**

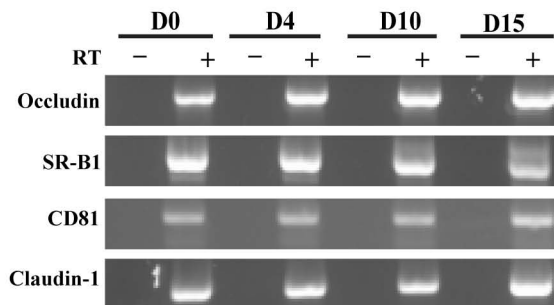

**A**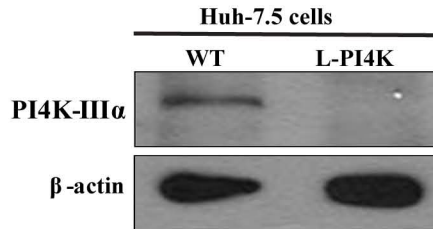**B**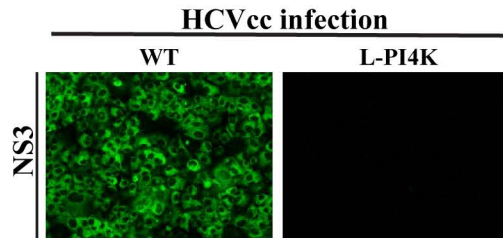**C**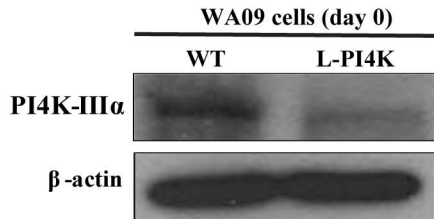**D**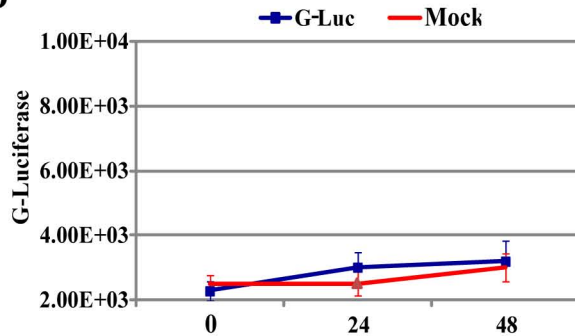

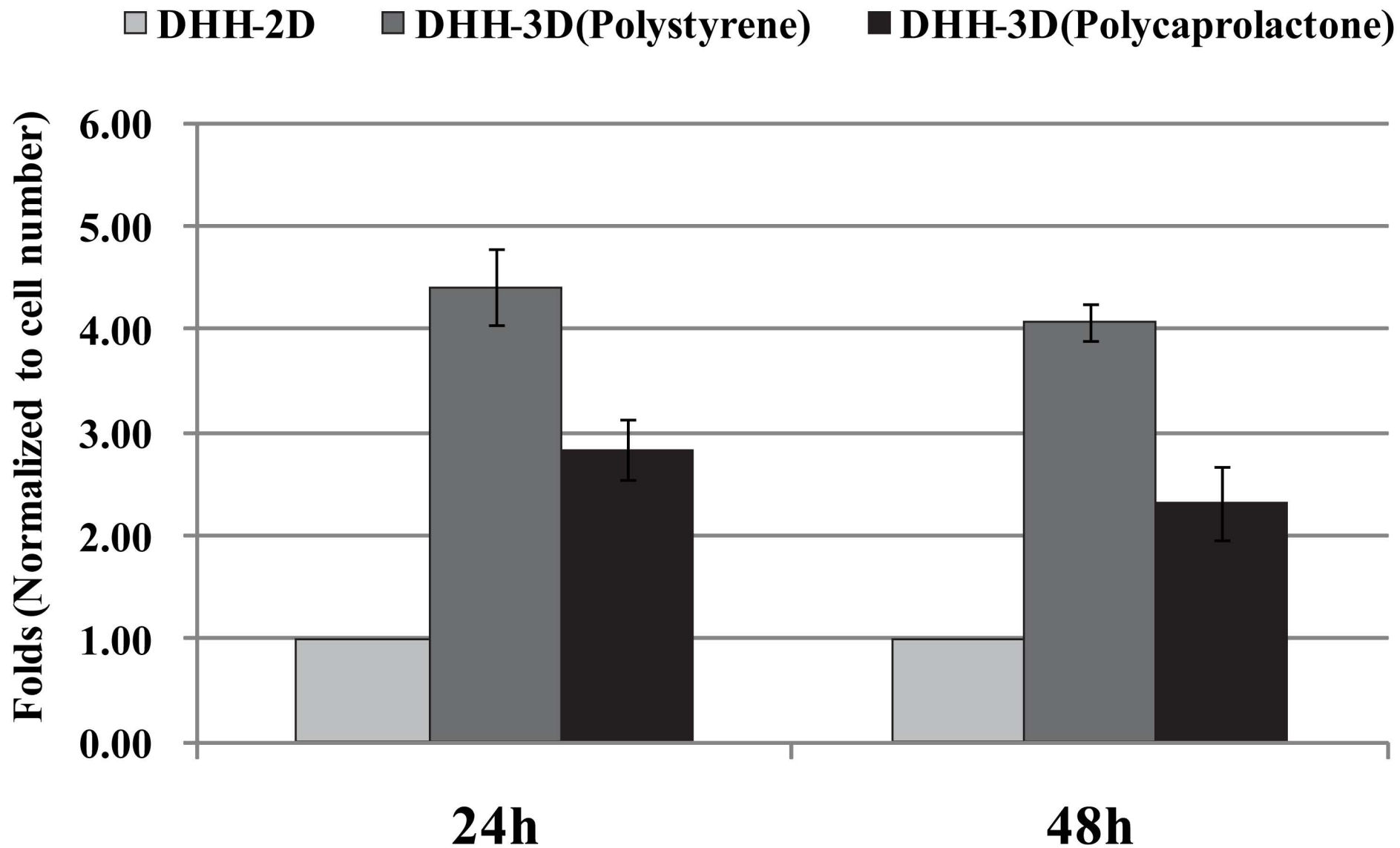

Supplement: Text S1 — Supplemental Figures 1 – 5 . Figure S1. Expression of mRNAs of hepatic markers during differentiation. Expression levels were normalized to GAPDH and the those of PHH was set to be 1. ASGR1: asialoglycoprotein receptor-1; CyP3A: cytochrome P450, family 3, subfamily A; PHH: primary human hepatocytes. Figure S2. Expression of mRNAs of HCV cofactors during hepatic differentiation. (A) Expression of EGFR and EphA2 mRNA in DHHs and PHHs. (B) Microarray heat map of expression levels of reported HCV cofactors in day-10 and day-7 cells. (C) Expression profile of HCV cofactors as represented by conventional RT-PCR and gel analysis. Figure S3. HCV receptor molecules expressed in stem cells. (A) Cell-surface staining of the four well-characterized receptors (CD81, SR-BI, Claudin-1, and Occludin) for HCV entry in both H9 and day-10 cells. (B) RT-PCR analysis of receptor expression during the hepatic differentiation process. Figure S4. PI4KIII knockdown in DHHs block HCV infection. (A) Suppression of PI4KIIIα by shRNA in Huh-7.5 cells. (B) PI4KIIIα KD efficiently blocked HCV infection in Huh-7.5 cells. (C) PI4KIIIα KD in H9 cells. (D) DHHs with PI4KIIIα KD were resistant to HCV infection. The cells were infected at day 13, and the luciferase activity was monitored for the next 48 h. Error bars represent standard deviations of replicate experiments. Figure S5. Increased infection efficiency of DHHs cultured in three-dimensional scaffolds. For the 3-D cultures, day-9 cells were seeded onto either polystyrene or polycaprolactone scaffolds, which were transferred to a new dish after adherence of the cells. Infections by Jc1/GLuc2A were performed at day 13, and luciferase assays in the next two days. The luciferase results were normalized to the cell numbers and then compared with those of the regular (2-D) cultures, which were set to be 100%. Error bars represent standard deviations of replicate experiments. (PDF) [file ppat.1002617.s002.pdf]
