# Supplementary material for: Productive Hepatitis C Virus Infection of Stem Cell-Derived Hepatocytes Reveals a Critical Transition to Viral Permissiveness during Differentiation
Source: PLoS Pathog. 2012 Apr 5;8(4):e1002617. doi: 10.1371/journal.ppat.1002617 (PMC3320597; doi:10.1371/journal.ppat.1002617)
Supplement: Table S1 — Antibody list. Name and source of the antibodies (suppliers and catalog numbers) used in this study are listed. (DOC) [file ppat.1002617.s003.doc]

Supplementary Table 1

**List of a**ntibodies used in this study.

| **Antigen** | **Provider** | **Catalog Number** |
| --- | --- | --- |
| HCV Core, NS3, and NS5A | BioFront Technologies | 3D11/4F5/2H1/4F9, 2E3, 7B5 |
| Human Oct-4 | Santa Cruz Biotechnology | sc-5279 |
| Human CXCR4 | NIH AIDS Regents Program | MAB172 |
| ALB, FLAG | Sigma Aldrich | SAB3300097, F7525 |
| SR-BI | Novus Biologicals | NB400-101 |
| Claudin-1, Cytokeratin-7 | Invitrogen | 374900, 18-0234 |
| CD81 | BD Pharmingen | 555675 |
| Occludin | Abcam | ab31721 |
| Human α-fetoprotein | DAKO | ABIN370517 |
| Human DDX-3 | Dr. Robin Reed (Harvard Medical School). | n.a |
| EGFR, EphA2 | Thermo Scientific | MA5-15284, PA1-1110 |
| PI4KIIIα | Cell Signaling Technology | 4902 |
| Cyclophilin A | Biomol Enzo Life Sciences | BML-SA296-0100 |
